# Comparative Study of Intestinal Microbiota Composition of Six Edible Fish Species

**DOI:** 10.3389/fmicb.2021.760266

**Published:** 2021-12-07

**Authors:** Tamir Ofek, Maya Lalzar, Sivan Laviad-Shitrit, Ido Izhaki, Malka Halpern

**Affiliations:** ^1^Department of Evolutionary and Environmental Biology, Faculty of Natural Sciences, University of Haifa, Haifa, Israel; ^2^Central Fish Health Laboratory, Fishery and Aquaculture Department, Ministry of Agriculture and Rural Development, Nir David, Israel; ^3^Bioinformatics Service Unit, University of Haifa, Haifa, Israel; ^4^Department of Biology and Environment, Faculty of Natural Sciences, University of Haifa, Oranim, Kiryat Tiv’on, Israel

**Keywords:** edible fish, intensive freshwater aquaculture, trophic level, *Cetobacterium*, microbiota composition

## Abstract

Intensive freshwater aquaculture in the Spring Valley, Israel, is implemented mainly in earthen fishponds and reservoirs that are stocked with a variety of edible fish species. Here we sampled six different healthy fish species from these intensive aquacultures. The fish were hybrid striped bass, European bass, red drum (all carnivores), hybrid tilapia, flathead grey mullet (both herbivores), and common carp (an omnivore). Significant differences were found among the intestinal microbiota of the six studied fish species. The microbiota composition diversity was strongly related to the trophic level of the fish, such that there was a significant difference between the carnivore and the herbivore species, while the omnivore species was not significantly different from either group. The most abundant genus in the majority of the fishes’ intestinal microbiota was *Cetobacterium*. Furthermore, we found that beside *Cetobacterium*, a unique combination of taxa with relative abundance >10% characterized the intestine microbiota of each fish species: unclassified *Mycoplasmataceae*, *Aeromonas*, and *Vibrio* (hybrid striped bass); *Turicibacter* and *Clostridiaceae* 1 (European bass); *Vibrio* (red drum); *ZOR0006*—*Firmicutes* (hybrid tilapia); unclassified *Mycoplasmataceae* and unclassified *Vibrionaceae* (flathead grey mullet); and *Aeromonas* (common carp). We conclude that each fish species has a specific bacterial genera combination that characterizes it. Moreover, diet and the trophic level of the fish have a major influence on the gut microbiota of healthy fish that grow in intensive freshwater aquaculture.

## Introduction

More than half of vertebrates are represented by fish, which include enormous ecological diversity and possess distinguished intestinal tract features. Lately, class of fish has become relevant for studies that examine the association of hosts and their microorganisms (Talwar et al., 2018). Most of the fish species living today are teleosts, which possess, like all vertebrates, complex gut microbiota as well as immunological and physiological features. The digestive tract of teleosts, like mammals, consists of a pancreas, liver, gallbladder, and intestine which develop similarly to the way the rostral gut develops to the hindgut and midgut (Lescak and Milligan-Myhre, 2017).

A fish’s gut provides niches for adherence, colonization, and proliferation of pathogenic, mutualistic, and benign commensal microbial species, which affect many immunological and physiological host functions. The fish gut microbial community changes with the host’s developmental stage (Giatsis et al., 2014). Fish size may be related to gut microbiota composition within the same species (Gadoin et al., 2021). The fish’s intestine is able to harbor 10^7^ to 10^11^ bacteria for each gram of intestinal content (Navarrete et al., 2012). Seasonal dynamics may influence the fish’s intestinal bacterial load and composition (Al-Harbi and Naim Uddin, 2004). Intestine bacteria can be autochthonous species, which are attached to the intestinal mucosa, or allochthonous species that do not attach because of competition with the mucous attached bacteria or because they do not have the ability to attach (Dehler et al., 2017). Environmental conditions, trophic level and/or feeding behavior, and host specific characteristics are the three main factors governing gut microbiota community structure (Talwar et al., 2018).

Most of the aquaculture in Israel is implemented by polyculture in reservoirs and earthen fishponds; therefore, such water impoundments are stocked with a combination of tilapia hybrids (*Oreochromis aureus* × *O. niloticus*), common carp (*Cyprinus carpio*), flathead grey mullet (*Mugil cephalus*), and silver carp (*Hypophthalmichthys molitrix*). Some farms have also added red drum (*Sciaenops ocellatus*), grass carp (*Ctenopharyngodon idella*), and a hybrid silver carp–bighead carp cross (*H. molitrix* × *H. nobilis*) (Neori et al., 2017). Other farms stocked hybrid striped bass (*Moron saxatilis* × *M. chrysops*) (Dunning and Daniels, 2001) and European bass (*Dicentrarchus labrax*) (Roll et al., 2007). As far as we know, the microbiota of these species in an intensive freshwater aquaculture has not been studied before.

Here we aimed at understanding the effect of the trophic level on the intestinal microbiota of fish. Specifically, we addressed the question: what is the effect of fish diet, size, and season on the intestinal microbiota composition, diversity, and richness? To answer this question, we studied the intestinal microbiota of six fish species: hybrid striped bass (*Moron saxatilis* × *Moron chrysops*), European bass (*Dicentrarchus labrax*), red drum (*Sciaenops ocellatus*) (all carnivores), hybrid tilapia (*Oreochromis aureus* × *O. Niloticus*), flathead grey mullet (*Mugil cephalus*) (both herbivores), and common carp (*Cyprinus carpio*) (an omnivore). The fish were sampled from intensive freshwater aquacultures. Our results showed that diet has a major role on the intestine microbiota composition of the fish species. The results of this study provide basic data of the microbiota composition of healthy aquaculture fish. These data may help develop molecular monitoring tools for assessing fish health conditions.

## Materials and Methods

### Ethics Statement

All methods were performed in accordance with relevant guidelines and regulations (Johansen et al., 2006). All fish samples were collected from fish that were brought regularly for health examination to the Central Fish Health Laboratory (Fishery Department, Ministry of Agriculture and Rural Development) located at kibbutz Nir David, Israel. All experimental procedures and animal care were approved by the Committee of Animal Experimentation of the University of Haifa (permit 638/19).

### Fish Samples

Six different species of edible fish (*n* = 81) were sampled from fishponds that are located in the Spring Valley, Israel (longitude 35.516899, latitude 32.508246). Sampling took place between February 2018 and March 2019 and covered all four seasons in the Spring Valley (winter: Dec–Feb, spring: Mar–Apr, summer: May–Oct, and autumn: November). The following fish species were sampled: hybrid striped bass (*Moron saxatilis* × *Moron chrysops*; *n* = 17); European bass (*Dicentrarchus labrax*; *n* = 10); red drum (*Sciaenops ocellatus*; *n* = 9); hybrid tilapia (most of the tilapia farmed in Israel are hybrids of *Oreochromis aureus* males and *Oreochromis niloticus* females; *n* = 15); flathead grey mullet (*Mugil cephalus*; *n* = 17); and common carp (*Cyprinus carpio*; *n* = 13). Fish were divided by weight into two groups, small fish (<100 g) that usually grow in small fishponds for the “training” stage and big fish (>100 g) that are actively transferred by the fisherman to a larger fishpond or a larger reservoir for the fattening stage (European bass and red drum samples included only big fish). Fish sampling details are specified in Supplementary Table 1.

### Fish Feed

Fish feed was primarily pellets manufactured by two main feed companies, Zemach Extrufeed Aqua^1^ and Raanan Fish Feed^2^. Fish food pellets contain different protein and fat percentages for each species and are usually made from poultry by-products, cereals and cereal by-products, seed oils and their by-products, and fish oil. Predator fish pellets are added fishmeal due to higher protein requirements of carnivorous species. Flathead grey mullet do not have special feed; they are only raised in polyculture and eat the feed of the primary fish in the fishpond/reservoir. For more details, see Supplementary Table 2.

### Intestine Content Sampling

Samples of intestine from healthy fish were taken separately in aseptic conditions with surgical instruments that were soaked in ethanol (70%) and burned in flame. The samples were transferred into 2-ml sterile test tubes (three tubes for each sample) containing 750 μl of absolute ethanol and then kept at −20°C until DNA extraction.

### DNA Extraction

To obtain DNA without ethanol residues, the tubes with the intestine samples were centrifuged for 30 min at maximum speed, and the ethanol was removed from the tubes. DNA was extracted from the samples as described previously by Laviad-Shitrit et al. (2017), using a DNA isolation kit (DNeasy Blood and Tissue, Qiagen, Hilden, Germany) according to the manufacturer’s instructions with minor modifications. The extracted DNA samples were stored at −20°C.

### Generation of the 16S rRNA Gene Library

A set of primers was used to amplify the V4 variable region of the 16S rRNA gene: CS1_515F (ACACTGACGACATGGTTCTA CAGTGCCAGCMGCCGCGGTAA) and CS2_806R (TACG GTAGCAGAGACTTGGTCTGGACTACHVGGGTWTCTAAT) (Sigma-Aldrich, Rehovot, Israel) (Caporaso et al., 2012). PCR amplification was performed using the EmeraldAmp MAX HS PCR Master Mix (Takara Bio Inc., Otsu, Shiga, Japan). The primers contained 5′ common sequence tags (also known as common sequence 1 and 2, CS1 and CS2). Amplicons were created using a two-stage “targeted amplicon sequencing (TAS),” as described previously by Naqib et al. (2018). PCR was performed as described by Sela et al. (2020). Sterile DNA-free water was used as a negative control for DNA extraction and PCR amplification to verify that there was no contamination.

### Illumina MiniSeq Sequencing

Subsequently, a second PCR amplification was performed in 10-μl reactions in 96-well plates. A master mix for the entire plate was made using the MyTaq HS 2X master mix. Each well received a separate primer pair with a unique 10-base barcode, obtained from the Access Array Barcode Library for Illumina (Fluidigm, South San Francisco, CA, United States; Item# 100-4876). These Access Array primers contained the CS1 and CS2 linkers at the 3′ ends of the oligonucleotides. The conditions for the second PCR and the procedure of the Illumina sequencing were performed as was described in Sela et al. (2020). The second PCR, library preparation, pooling, and sequencing were performed at the University of Illinois at Chicago Sequencing Core (UICSQC) within the Research Resources Center (RRC).

### Sequence Analysis

In total, 324 files in fastq format were generated, corresponding to 81 samples (four files for each sample), with two paired-end sequences each. Data were examined with the fastQC program^3^. All the samples were of high quality in both directions of sequencing. Sequence data were analyzed using the DADA2 pipeline (Callahan et al., 2016). A detailed description of the data analysis is described in Laviad-Shitrit et al. (2021). Following the data analysis, both runs were merged by sample, and amplicon sequence variants (ASVs) of non-bacterial origin (Archaea, chloroplast, mitochondria, and unclassified) were filtered out.

Raw sequence data were submitted to the National Center for Biotechnology Information Sequence Read Archive^4^ under the BioProject accession number PRJNA748160.

### Statistical Analysis

All statistical analyses were performed in R version 3.6.2 (R Core Team, 2019) unless otherwise specified. Data were subsampled to 20,000 sequences per sample and normalized before the statistical analysis was performed. Rarefaction curves were calculated using MicrobiomeAnalyst (Chong et al., 2020). Alpha diversity was calculated using the Simpson coefficient and compared among fish species by Kruskal–Wallis test and Dunn test for *post hoc*, using Benjamini–Hochberg correction for false discovery rate. Beta diversity, fish size, and the interaction between them were calculated by non-metric multidimensional scaling (NMDS) (Bray–Curtis dissimilarity) and pairwise ADONIS test with Benjamini–Hochberg correction for false discovery rate. ASV linear discriminant analysis (LDA) scores were calculated by LDA effect size (LEfSe).

## Results

Six fish species (*n* = 81) were sampled from fish ponds located in the Spring Valley, Israel (Supplementary Table 1). The fish species included three carnivores: hybrid striped bass, European bass and red drum, two herbivores: flathead grey mullet and hybrid tilapia, and one omnivore: common carp.

The fish intestinal microbiota was studied using 16S rRNA gene sequencing. In total, 4,796,546 quality reads were generated with an average of 59,216 reads per sample and an overall total of 2,290 ASVs. Rarefaction curves of each sample reached an asymptotic level, suggesting that our sampling efforts were sufficient to obtain a full estimate of ASV richness (Supplementary Figure 1).

The intestine microbiota composition of the fish species was significantly different between the six fish species and between the two fish sizes (small fish *vs*. big fish). Moreover, the interaction between these factors, the fish trophic level, and the fish sampling season were also significant (Table 1).

**TABLE 1:** ADONIS test showing that there was a significant difference between the microbiota composition of the fish and different factors.

Factor	*df*	*F*	*R* ^2^	*p*
Fish species	5	4.91	0.230	0.001
Fish size	1	2.95	0.027	0.003
Fish species × fish size	3	2.71	0.076	0.001
Fish trophic level	2	1.95	0.047	0.003
Fish sampling season	3	1.80	0.065	0.003

**FIGURE 1:**
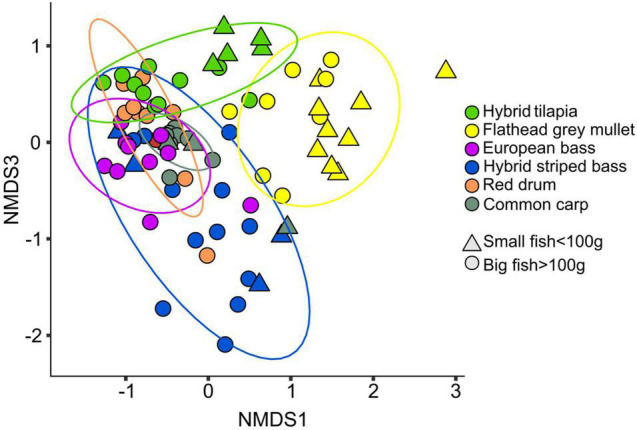
NMDS (Bray–Curtis dissimilarity) plot for the intestinal microbiota composition (stress value <0.2, *K* = 3) of small (<100 g) and big (>100 g) fish from the six different species. Significant differences were found between the microbiota composition of the different fish species (Table 1 and Supplementary Table 3).

Non-metric multidimensional scaling results (Figure 1) based on pairwise ADONIS test (with Benjamini–Hochberg correction for false discovery rate, Supplementary Table 3) revealed that the microbiota composition of small and big fish (from the same species) was significantly different in the hybrid tilapia and the common carp. In the small fish, there was a significant difference between the microbiota composition of hybrid striped bass and flathead grey mullet, hybrid striped bass and hybrid tilapia, and between flathead grey mullet and hybrid tilapia. The microbiota composition of the big fish was significantly different between all fish species, except for the red drum and hybrid tilapia. All pairwise ADONIS test results are specified in Supplementary Table 3. The NMDS (Figure 1) demonstrated that the common carp samples were spread in a narrow range, which may indicate a low variability among the intestinal microbiota composition of the samples. The intestinal microbiota composition of the flathead grey mullet samples was significantly different from all the other fish species (except for the common carp in the small fish). Moreover, most of the flathead grey mullet samples were situated far from any of the other fish species samples (Figure 1), suggesting that the intestine microbiota composition of this species was unique. The hybrid striped bass samples were spread across a wide area, which may indicate high variability among the intestinal microbiota of the samples. The microbiota composition of the hybrid tilapia was similar to that of the red drum as denoted by a large overlap in the samples of these species (Figure 1).

**FIGURE 2:**
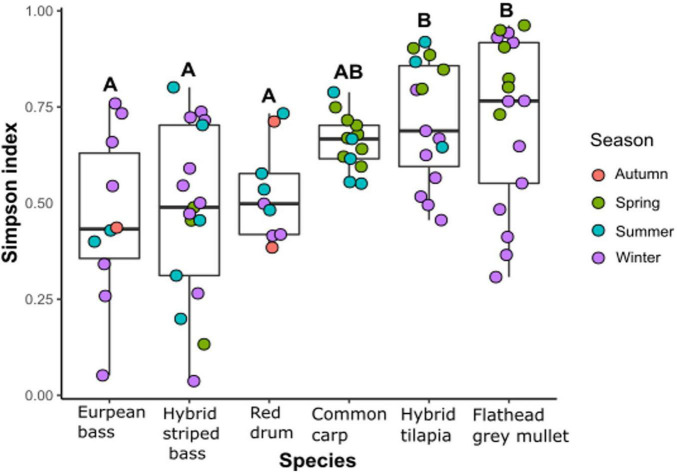
α-Diversity (Simpson index) of the intestinal microbiota composition from the six different fish species (Kruskal–Wallis Chi-squared = 20.562, df = 5, *p* < 0.001) by seasons. Letters indicate significant differences based on Dunn test for *post hoc* using Benjamini–Hochberg correction for false discovery rate.

To examine the effect of fish species on bacterial diversity, the alpha diversity (Simpson index) of the intestinal microbiota was calculated. The Simpson index differed significantly among the six fish species (Figure 2). Moreover, there was a significant difference between the carnivore species, which had the lowest species richness, and the herbivore species, which had the highest species richness. The intestinal microbiota composition of the common carp, an omnivorous fish, was not significantly difference from the other species (Figure 2). The Simpson index of the intestinal microbiota composition was also significantly different among the sampled seasons (Kruskal–Wallis chi-squared = 9.96, df = 3, *p* < 0.05). However, according to the Dunn test results, there was only a significant difference between winter and spring, probably because of the herbivore species samples that showed higher Simpson index values in spring than in winter.

### The Microbiota Community Composition of Fish Intestines

**FIGURE 3:**
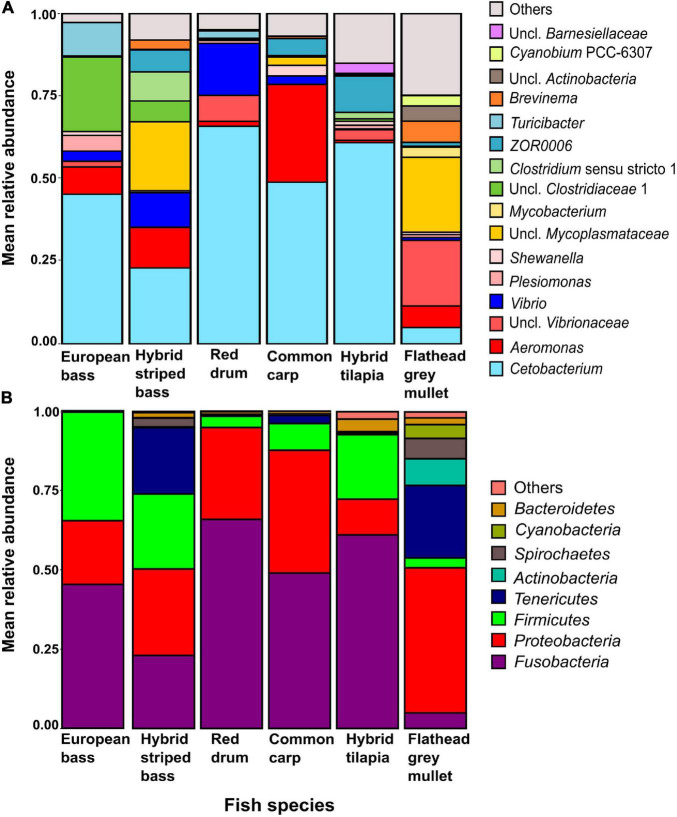
Taxa mean relative abundances up to the genus **(A)** and at the phylum **(B)** levels for the intestinal microbiota in the studied fish species. Uncl., unclassified.

At the genus level (Figure 3A), *Cetobacterium* was the most dominant in five of the six fish species, from 23% in the hybrid striped bass to nearly 66% in the red drum. Its abundance in the flathead grey mullet was relatively lower (4.9%). Other dominant taxa were an unclassified *Mycoplasmataceae* with nearly 23% in the flathead grey mullet and nearly 21% in the hybrid striped bass, and *Aeromonas* with nearly 30% in the common carp and 12% in the hybrid striped bass (Figure 3A, Table 2); an unclassified *Clostridiaceae* 1 with about 22% in the European bass, and *Vibrio* with nearly 16% mean relative abundance in the red drum, and about 10% in the hybrid striped bass (Figure 3A, Table 2 and Supplementary Table 4). At the phylum level (Figure 3B), the *Fusobacteria* was the most dominant phylum in four out of the six species with more than 45% mean relative abundance in the European bass and up to nearly 66% in the red drum. *Proteobacteria* was the most dominant in flathead grey mullet and in hybrid striped bass with more than 45% and nearly 27% mean relative abundance, respectively. Moreover, *Proteobacteria* was the second most dominant phylum in common carp and red drum with nearly 39 and 29% mean relative abundance, respectively. *Firmicutes* was the second most dominant phylum in European bass, hybrid tilapia, and hybrid striped bass, with nearly 34, 24, and 20% mean relative abundance, respectively. *Tenericutes* was the second most dominant phylum in flathead grey mullet with nearly 23% mean relative abundance and the fourth most dominant in hybrid striped bass with nearly 23% (Figure 3B and Supplementary Table 4).

**TABLE 2:** Highly dominant intestinal microbiota taxa, classified up to the genus level with 10% or higher relative abundance in at least one fish species.

Taxon	Class	European bass	Hybrid striped bass	Red drum	Common carp	Hybrid tilapia	Flathead grey mullet
*unclassified Mycoplasmataceae*	*Mollicutes*	0.0	**20.9**	0.0	2.5	0.0	**22.7**
*Aeromonas*	*Gammaproteobacteria*	8.3	**12.2**	1.5	**29.6**	0.7	6.6
*unclassified Vibrionaceae*	*Gammaproteobacteria*	1.8	0.1	7.9	0.0	3.2	**19.9**
*Vibrio*	*Gammaproteobacteria*	3.1	**10.4**	**15.8**	2.5	0.3	0.8
*Cetobacterium*	*Fusobacteria*	**45.3**	**23.0**	**65.9**	**49.0**	**60.9**	4.9
*ZOR0006*	*Erysipelotrichia*	0.0	6.7	0.2	5.2	**11.0**	1.1
*Turicibacter*	*Erysipelotrichia*	**10.1**	0.2	2.2	0.0	0.3	0.0
*Clostridiaceae* 1	*Clostridia*	**22.5**	6.3	0.0	0.1	0.7	0.1

*Taxa with the abundance of more than 10% are marked in bold.*

### Linear Discriminant Analysis

**FIGURE 4:**
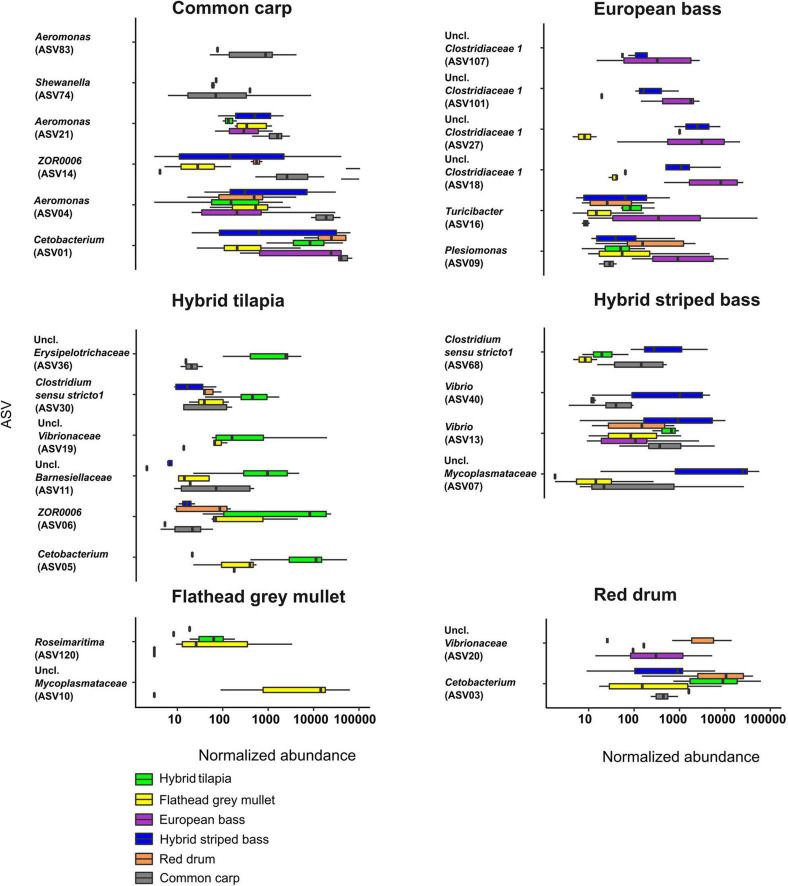
Normalized abundance of six different fish species intestinal ASVs. The ASVs that were chosen (two to six ASVs for each fish species) were those with the highest LDA score by LEfSe analysis. Uncl., unclassified.

To identify which ASVs contributed significantly to the variation in the microbiota of the different fish species, we used the LDA effect size (LEfSe) to present the normalized abundance of the ASVs for each fish species (Figure 4). In common carp, *Cetobacterium* (ASV01) was the genus with the highest abundance of all discriminant ASVs, and *Aeromonas* was represented by three ASVs (ASV04, ASV21, and ASV83). In the European bass, the dominant genus was unclassified *Clostridiaceae* 1, represented by four ASVs (ASV18, ASV27, ASV101, and ASV107). The flathead grey mullet had only two discriminant ASVs, ASV10 that belonged to an unclassified genus in the *Mycoplasmataceae* family and ASV120 from the genus *Roseimaratima* that was very rare across all other samples. The red drum had only two discriminant ASVs as well, which included ASV03 (*Cetobacterium*) and ASV20 (an unclassified *Vibrionaceae*). In hybrid striped bass, an ASV from an unclassified genus of *Mycoplasmataceae* (ASV07) had the highest normalized abundance, and *Vibrio* was the dominant genus, represented by two ASVs (ASV13 and ASV40). In the hybrid tilapia, *Cetobacterium* (ASV05) and ASV06 from the genus *ZOR0006* had the highest normalized abundance.

## Discussion

Here we examined the intestinal microbiota composition of six different edible fish species from intensive freshwater aquaculture. Previous studies on fishes from a variety of habitats showed that fish gut microbiota composition and diversity are influenced by diet and feeding habit (Wang et al., 2017). In the current study, the intestinal microbiota composition was significantly different between the six fish species (Table 1). When the fish were divided into two groups by size, there was a significant difference between the microbiota of all the six fish species, except for the red drum and the hybrid tilapia, when considering only big fish (Figure 1 and Supplementary Table 1). When making pairwise comparisons of big and small fish of the same species, only two out of the four sampled small species (common carp and hybrid tilapia) showed significant differences between the microbiota composition. Gadoin et al. (2021) found that only two of three species of tuna fish (yellowfin and bigeye) from the eastern Atlantic Ocean had significant differences between the microbiota composition of small and big fish within the species, while the third species (skipjack) did not. They explained that the reason for this pattern was likely steady feeding behavior of the third species during its lifetime as opposed to differential, age-related feeding habit in the other two species (Gadoin et al., 2021).

We found a strong association between the diversity of the intestinal microbiota and the three different trophic levels to which the fish belonged. This was also reflected by significant differences between the intestinal microbiota of the herbivore and the carnivore groups. When the gut microbiota composition of eight fish species from four trophic levels was studied (Liu et al., 2016), significant differences were also found between the diversity of the gut microbiota composition of herbivore *vs*. carnivore fish species. Moreover, it was found that the diversity of the gut microbiota composition of the omnivore fish species (one of the two species was common carp) was not significantly different from the diversity of the microbiota of the other two trophic levels (Liu et al., 2016). Similar results were found in the current study, which may point at a strong impact of the fish trophic level on the diversity of the fish intestinal microbiota.

Seasonal changes in the fish intestinal microbiota were significant only between winter and spring. Al-Harbi and Naim Uddin (2004) found seasonal variation in the intestinal microbiota of the hybrid tilapia from earth ponds in Saudi Arabia. Hagi et al. (2004) found seasonal changes in intestinal lactic acid bacteria of three carp species and channel catfish from Lake Kasumigaura in Japan. However, Hovda et al. found no evidence for seasonal differences between the gut microbiota of farmed Atlantic salmon (Hovda et al., 2012).

*Cetobacterium* was found in all of the fish species and was the most abundant genus in five out of the six studied species (Figure 3A). *Cetobacterium* was also reported as the most dominant genus in the intestinal microbiota of some freshwater fish such as *Arapaima gigas* (Ramírez et al., 2018), *Lepomis macrochirus*, *Micropterus salmoides*, and *Ictalurus punctatus* (Larsen et al., 2014), a variety of cichlid species (Baldo et al., 2015, 2017, 2019), and the common carp (van Kessel et al., 2011). *Cetobacterium* that was isolated from the intestine of a freshwater fish was found to produce vitamin B12 (Tsuchiya et al., 2008). Recently, Wang et al. (2021) found that an increase in acetate producing *Cetobacterium somerae* contributed to glucose homeostasis and improved fish carbohydrate utilization.

The beta-diversity (Figure 1) of the fish species and the mean relative abundances at the genus level (Figure 3A) revealed that the microbiota of big common carp was significantly different from all the other fish species, regardless of size. Further, this subgroup had low variation among samples. This was also reflected by the dominance of *Cetobacterium* and *Aeromonas* (48.9 and 29.6%, respectively). Eichmiller et al. (2016), who studied the intestinal microbiota composition of common carps that were sampled from freshwater rivers and lakes in the United States, found that their microbiota composition was comprised of *Cetobacterium* sp. (23.8%) and the order *Aeromonadales* (10.0%) (Eichmiller et al., 2016).

The flathead grey mullet intestinal microbiota composition was significantly different from all other fish species, and the samples were spread across a wide range with almost no overlap with the other species samples (Figure 1). It seems that this phenomenon might be explained by the fact that nearly 25% of this species microbiota composition included many ASVs with relatively low abundances. *Mycoplasmataceae* abundance was 23%. This ASV was not present or was present at very low abundances in the other fish species (Figure 4). Similarly, Le and Wang (2020) found that flathead grey mullet that were sampled from marine water also had high abundance of *Mycoplasmataceae*.

The red drum and the big hybrid tilapia were the only fish groups whose intestinal microbiota were not significantly different. This may be explained by the fact that *Cetobacterium* abundances in their microbiota were 66 and 61%, respectively.

In general, three *Cetobacterium* ASVs (ASV01, ASV03, and ASV05) had the highest relative abundances (Figure 4) in common carp, red drum, and hybrid tilapia, respectively. Moreover, each fish had a specific bacterial fingerprint. The combination of *Cetobacterium*, an unclassified *Mycoplasmataceae*, *Aeromonas*, and *Vibrio* may characterize the intestinal microbiota composition of the hybrid striped bass, while the European bass can be characterized by the combination of *Cetobacterium*, *Clostridiaceae* 1, and *Turicibacter*. *Cetobacterium* and *Vibrio* characterize the red drum species, and in the case of hybrid tilapia, the combination of *Cetobacterium* and *ZOR0006* (*Firmicutes*) can be considered typical. An unclassified *Mycoplasmataceae* and an unclassified *Vibrionaceae* characterize the flathead grey mullet, while *Cetobacterium* and *Aeromonas* may characterize the common carps’ microbiota (Table 2).

## Conclusion

The main aim of this study was to define the intestine microbiota composition of six healthy edible fish species from intensive freshwater aquaculture. Significant differences were found between the intestine microbiota composition of the different fish species, in the different sampling seasons, and between fish sizes. The diversity of the intestinal microbiota composition was strongly related to the trophic level of the fish. *Cetobacterium* was present in all the fish species, and it was a major candidate in five out of the six species. *Cetobacterium* probably plays a beneficial role in biochemical processes in the fish gut. Some ASVs significantly contributed to the variation in the microbiota composition of the different fish species. In fact, it appears that each fish species can be characterized by the presence of specific bacterial genera combination in their intestine. Further research is needed to investigate the relationship between the microbiota composition of fish in intensive freshwater aquaculture and fish health.

## Data Availability Statement

The datasets presented in this study can be found in online repositories. The names of the repository/repositories and accession number(s) can be found below: https://www.ncbi.nlm.nih.gov/, PRJNA748160.

## Ethics Statement

The animal study was reviewed and approved by Committee of Animal Experimentation of the University of Haifa (permit 638/19).

## Author Contributions

TO, II, and MH conceived and designed the experiments. II and MH contributed reagents and materials and analysis tools. TO and SL-S performed the experiments. TO and ML analyzed the data. TO wrote the manuscript. All authors discussed the results and reviewed and commented the manuscript and approved the submitted version.

## Conflict of Interest

The authors declare that the research was conducted in the absence of any commercial or financial relationships that could be construed as a potential conflict of interest.

## Publisher’s Note

All claims expressed in this article are solely those of the authors and do not necessarily represent those of their affiliated organizations, or those of the publisher, the editors and the reviewers. Any product that may be evaluated in this article, or claim that may be made by its manufacturer, is not guaranteed or endorsed by the publisher.

## Footnotes

1
https://zemach-extrufeed.co.il

2
https://raanan-fishfeed.com

3
https://www.bioinformatics.babraham.ac.uk/projects/fastqc

4
https://www.ncbi.nlm.nih.gov/bioproject/

## References

[B1] Al-Harbi A. H. Naim Uddin M. (2004). Seasonal variation in the intestinal bacterial flora of hybrid tilapia (*Oreochromis niloticus×Oreochromis aureus*) cultured in earthen ponds in Saudi Arabia. *Aquaculture* 229 37–44. 10.1016/S0044-8486(03)00388-0

[B2] Baldo L. Pretus J. L. Lluís Riera J. Musilova Z. Bitja Nyom A R. Salzburger W. (2017). Convergence of gut microbiotas in the adaptive radiations of African cichlid fishes. *ISME J.* 11 1975–1987. 10.1038/ismej.2017.62 28509910 PMC5560477

[B3] Baldo L. Riera J. L. Salzburger W. Barluenga M. (2019). Phylogeography and ecological niche shape the cichlid fish gut microbiota in central American and African lakes. *Front. Microbiol.* 10:2372. 10.3389/fmicb.2019.02372 31681230 PMC6803461

[B4] Baldo L. Riera J. L. Tooming-Klunderud A. Mar Albà M. Salzburger W. (2015). Gut microbiota dynamics during dietary shift in eastern African cichlid fishes. *PLoS One* 10:e0127462. 10.1371/journal.pone.0127462 25978452 PMC4433246

[B5] Callahan B. J. McMurdie P. J. Rosen M. J. Han A. W. Johnson A. J. A. Holmes S. P. (2016). DADA2: high-resolution sample inference from illumina amplicon data. *Nat. Methods* 13 581–583. 10.1038/nmeth.3869 27214047 PMC4927377

[B6] Caporaso J. G. Lauber C. L. Walters W. A. Berg-Lyons D. Huntley J. Fierer N. (2012). Ultra-high-throughput microbial community analysis on the illumina Hiseq and Miseq platforms. *ISME J.* 6 1621–1624. 10.1038/ismej.2012.8 22402401 PMC3400413

[B7] Chong J. Liu P. Zhou G. Xia J. (2020). Using microbiomeanalyst for comprehensive statistical, functional, and meta-analysis of microbiome data. *Nat. Protoc.* 15 799–821. 10.1038/s41596-019-0264-1 31942082

[B8] Dehler C. E. Secombes C. J. Martin S. A. M. (2017). Environmental and physiological factors shape the gut microbiota of Atlantic salmon parr (*Salmo salar l*.). *Aquaculture* 467 149–157. 10.1016/j.aquaculture.2016.07.017 28111483 PMC5142738

[B9] Dunning R. Daniels H. (2001). Hybrid striped bass production in ponds enterprise budget. *South. Reg. Aquac. Center* 3000 1–6.

[B10] Eichmiller J. J. Hamilton M. J. Staley C. Sadowsky M. J. Sorensen P. W. (2016). Environment shapes the fecal microbiome of invasive carp species. *Microbiome* 4:44. 10.1186/s40168-016-0190-1 27514729 PMC4981970

[B11] Gadoin E. Durand L. Guillou A. Crochemore S. Bouvier T. D’orbcastel E. R. (2021). Does the composition of the gut bacteriome change during the growth of tuna? *Microorganisms* 9:1157. 10.3390/microorganisms9061157 34072252 PMC8229391

[B12] Giatsis C. Sipkema D. Smidt H. Verreth J. Verdegem M. (2014). The colonization dynamics of the gut microbiota in *Tilapia larvae*. *PLoS One* 9:e103641. 10.1371/journal.pone.0103641 25072852 PMC4114968

[B13] Hagi T. Tanaka D. Iwamura Y. Hoshino T. (2004). Diversity and seasonal change in lactic acid bacteria in the intestinal tract of cultured freshwater fish. *Aquaculture* 234 335–346. 10.1016/j.aquaculture.2004.01.018

[B14] Hovda M. B. Fontanillas R. Mcgurk C. Obach A. Thomas Rosnes J. (2012). Seasonal variations in the intestinal microbiota of farmed Atlantic salmon (*Salmo salar l*.). *Aquac. Res.* 43 154–159. 10.1111/j.1365-2109.2011.02805.x

[B15] Johansen R. Needham J. R. Colquhoun D. J. Poppe T. T. Smith A. J. (2006). Guidelines for health and welfare monitoring of fish used in research. *Lab. Anim.* 40 323–340. 10.1258/002367706778476451 17018205

[B16] Larsen A. M. Mohammed H. H. Arias C. R. (2014). Characterization of the gut microbiota of three commercially valuable warmwater fish species. *J. Appl. Microbiol.* 116 1396–1404. 10.1111/jam.12475 24529218

[B17] Laviad-Shitrit S. Lev-Ari T. Katzir G. Sharaby Y. Izhaki I. Halpern M. (2017). Great cormorants (*Phalacrocorax carbo*) as potential vectors for the dispersal of *Vibrio Cholerae*. *Sci. Rep.* 7:7973. 10.1038/s41598-017-08434-8 28801549 PMC5554209

[B18] Laviad-Shitrit S. Sharaby Y. Sela R. Thorat L. Nath B. B. Halpern M. (2021). Copper and chromium exposure affect chironomid Larval microbiota composition. *Sci. Total Environ.* 771:145330. 10.1016/j.scitotenv.2021.145330 33545485

[B19] Le M. H. Wang D. (2020). Structure and membership of gut microbial communities in multiple fish cryptic species under potential migratory effects. *Sci. Rep.* 10:7547. 10.1038/s41598-020-64570-8 32372020 PMC7200715

[B20] Lescak E. A. Milligan-Myhre K. (2017). Teleosts as model organsim to understand host microbe interactions. *J. Bacteriol.* 199 e00868–e816. 10.1128/JB.00868-16 28439034 PMC5512220

[B21] Liu H. Guo X. Gooneratne R. Lai R. Zeng C. Zhan F. (2016). The gut microbiome and degradation enzyme activity of wild freshwater fishes influenced by their trophic levels. *Sci. Rep.* 6:24340. 10.1038/srep24340 27072196 PMC4829839

[B22] Naqib A. Poggi S. Wang W. Hyde M. Kunstman K. Green S. J. (2018). Making and sequencing heavily multiplexed, high-throughput 16s ribosomal rna gene amplicon libraries using a flexible, two-stage PCR protocol. *Methods Mol. Biol.* 1783 149–169. 10.1007/978-1-4939-7834-2_7 29767361

[B23] Navarrete P. Magne F. Araneda C. Fuentes P. Barros L. Opazo R. (2012). PCR-TTGE analysis of 16S RRNA from rainbow trout (*Oncorhynchus mykiss*) gut microbiota reveals host-specific communities of active bacteria. *PLoS One* 7:e31335. 10.1371/journal.pone.0031335 22393360 PMC3290605

[B24] Neori A. Shpigel M. Guttman L. Israel A. (2017). Development of polyculture and integrated multi - trophic aquaculture (IMTA) in Israel: a review. *Isr. J. Aquac.* 69 1–19.

[B25] R Core Team (2019). *R: A Language and Environment for Statistical Computing.* Vienna: R Foundation for Statistical Computing.

[B26] Ramírez C. Coronado J. Silva A. Romero J. (2018). Cetobacterium is a major component of the microbiome of giant amazonian fish (Arapaima gigas) in ecuador. *Animals* 8:189. 10.3390/ani8110189 30352962 PMC6262583

[B27] Roll U. Dayan T. Simberloff D. Goren M. (2007). Characteristics of the introduced fish fauna of Israel. *Biol. Invasions* 9 813–824. 10.1007/s10530-006-9083-8

[B28] Sela R. Laviad-Shitrit S. Halpern M. (2020). Changes in microbiota composition along the metamorphosis developmental stages of *Chironomus transvaalensis*. *Front. Microbiol.* 11:586678. 10.3389/fmicb.2020.586678 33240240 PMC7677345

[B29] Talwar C. Nagar S. Lal R. Krishan Negi R. (2018). Fish gut microbiome: current approaches and future perspectives. *Indian J. Microbiol.* 58 397–414. 10.1007/s12088-018-0760-y 30262950 PMC6141390

[B30] Tsuchiya C. Sakata T. Sugita H. (2008). Novel ecological niche of cetobacterium somerae, an anaerobic bacterium in the intestinal tracts of freshwater fish. *Lett. Appl. Microbiol.* 46 43–48. 10.1111/j.1472-765X.2007.02258.x 17944860

[B31] van Kessel M. A. Dutilh B. E. Neveling K. Kwint M. P. Veltman J. A. Flik G. (2011). Pyrosequencing of 16s RRNA gene amplicons to study the microbiota in the gastrointestinal tract of carp (*Cyprinus carpio l*.). *AMB Express* 1:41. 10.1186/2191-0855-1-41 22093413 PMC3226434

[B32] Wang A. Zhang Z. Ding Q. Yang Y. Bindelle J. Ran C. (2021). Intestinal cetobacterium and acetate modify glucose homeostasis via parasympathetic activation in zebrafish. *Gut Microbes* 13 1–15. 10.1080/19490976.2021.1900996 33840371 PMC8043178

[B33] Wang A. R. Ran C. Ringø E. Zhou Z. G. (2017). Progress in fish gastrointestinal microbiota research. *Rev. Aquac.* 10 626–640. 10.1111/raq.12191

