# Supplementary material for: Comparative Study of Intestinal Microbiota Composition of Six Edible Fish Species
Source: Front Microbiol. 2021 Dec 7;12:760266. doi: 10.3389/fmicb.2021.760266 (PMC8689067; doi:10.3389/fmicb.2021.760266)
Supplement: Supplementary file 2 [file Data_Sheet_1.pdf]

Supplementary Material

Supplementary Figures

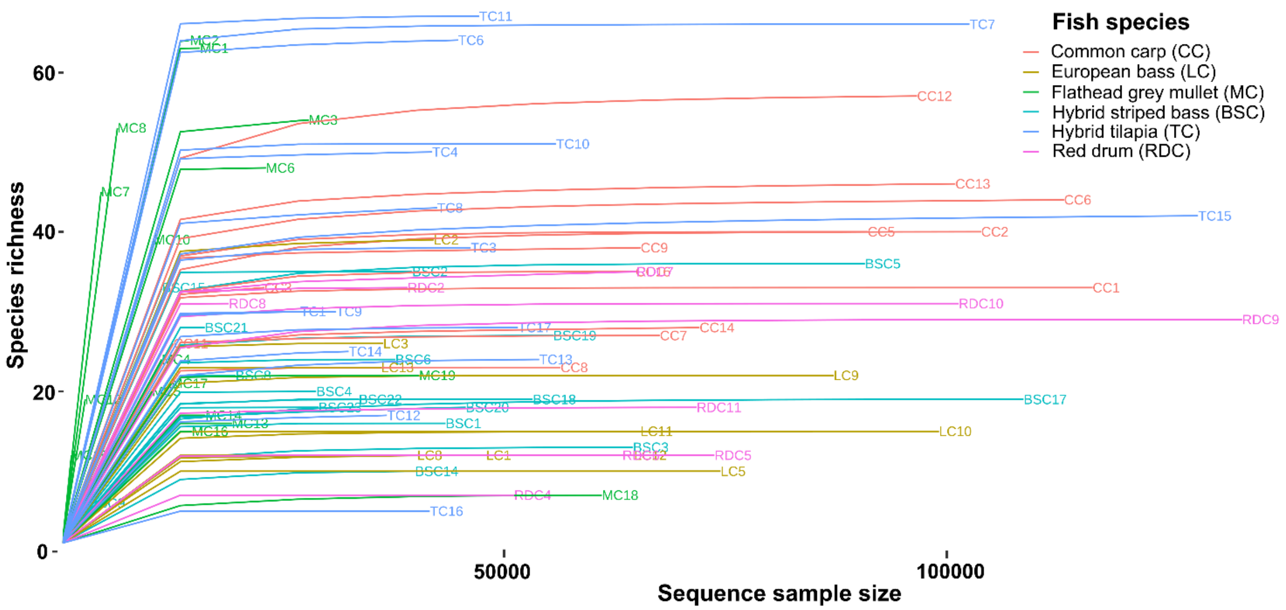

Supplementary Figure 1. Rarefaction curves representing the observed number of amplicon sequence variants (ASVs). Rarefaction curves present the increase in the number of the ASVs as a function of the sequencing depth for each sample.

Supplementary Tables

Supplementary Table 1. The details for the different fish species that were sampled in the current study (sampling season, average weight, and number of sampled fish).

Fish species & size	n	Sampling season	Average weight (gr)
Hybrid tilapia (small)	5	winter, spring	50
Hybrid tilapia (big)	10	summer, winter, spring	446
Flathead grey mullet (small)	9	winter, spring	40
Flathead grey mullet (big)	8	winter, spring	446
Hybrid striped bass (small)	4	winter, summer	46
Hybrid striped bass (big)	13	summer, winter, spring	603
Common carp (small)	3	summer	35
Common carp (big)	10	spring, summer	558
European bass (big)	10	autumn, winter, summer	162
Red drum (big)	9	autumn, winter, summer	487

Supplementary Table 2. Fish feed values for each fish species. Fish feed manufactured by Zemach Extrufeed Aqua (https://zemach-extrufeed.co.il) and/or Raanan Fish Feed (https://raanan-fishfeed.com).

Fish / Feed values	European bass (%)	Hybrid striped bass (%)	Red drum (%)	Common carp (%)	Hybrid tilapia (%)	Flathead grey mullet*
Crude protein	46.0	40.0	45.0	25.0 - 35.0	30.0 - 35.0	NA**
Crude fat	18.0	9.0	12.0	7.0 - 8.0	4.0 - 6.0	NA
Crude fiber	3.5	5.0	3.5	4.5 - 5.0	5.0 - 5.5	NA
Ash	10.0	8.5	9.0	8.5 - 9.0	7.0 - 9.0	NA
Moisture	10.0	9.5	9.5	9.5 - 10.0	9.5 - 10.0	NA
Calcium	2.0	2.2	2.0	1.80	1.2 - 1.7	NA
Phosphorus	1.0 - 1.5	1.0	1.0	0.8 - 1.3	1 - 1.2	NA

*Flathead grey mullet do not have special feed, they are only raised in polyculture and eat the feed of the primary fish in the pond/reservoir (usually hybrid tilapia, common carp or hybrid striped bass).

**NA, not available

Supplementary Table 3. Pairwise ADONIS test with Benjamini-Hochberg correction for false discovery rate test based on fish species and size.

Species	df	F	R^2	***p***	adjusted ***p*** (BH*)
**Fish size (small fish <100g *vs*. big fish >100g)**					
Hybrid striped bass	1	1.91	0.113	0.045	0.058
Common carp	1	5.10	0.317	0.023	0.033
Flathead grey mullet	1	1.07	0.067	0.374	0.382
Hybrid tilapia	1	8.20	0.387	0.001	0.002
**Fish species (small fish <100g)**					
Hybrid striped bass *vs*. Common carp	1	1.36	0.254	0.266	0.279
Hybrid striped bass *vs*. Flathead grey mullet	1	2.79	0.202	0.006	0.001
Hybrid striped bass *vs*. Hybrid tilapia	1	6.82	0.494	0.005	0.008
Common carp *vs*. Flathead grey mullet	1	1.52	0.144	0.074	0.088
Common carp *vs*. Hybrid tilapia	1	4.72	0.486	0.042	0.055
Flathead grey mullet *vs*. Hybrid tilapia	1	4.98	0.293	0.003	0.005
**Fish size (small fish <100g *vs*. big fish >100g**					
Hybrid striped bass *vs*. Common carp	1	4.42	0.167	0.001	0.002
Hybrid striped bass *vs*. European bass	1	1.99	0.086	0.025	0.035
Hybrid striped bass *vs*. Flathead grey mullet	1	2.34	0.110	0.001	0.002
Hybrid striped bass *vs*. Red drum	1	3.12	0.135	0.003	0.005
Hybrid striped bass *vs*. Hybrid tilapia	1	4.38	0.173	0.002	0.004
Common carp *vs*. European bass	1	4.99	0.208	0.001	0.002
Common carp *vs*. Flathead grey mullet	1	9.58	0.360	0.001	0.002
Common carp *vs*. Red drum	1	6.27	0.258	0.001	0.002
Common carp *vs*. Hybrid tilapia	1	10.08	0.346	0.001	0.002
European bass *vs*. Flathead grey mullet	1	2.80	0.149	0.001	0.002
European bass *vs*. Red drum	1	1.97	0.104	0.035	0.048
European bass *vs*. Hybrid tilapia	1	3.54	0.164	0.002	0.004
Flathead grey mullet *vs*. Red drum	1	3.99	0.210	0.001	0.002
Flathead grey mullet *vs*. Hybrid tilapia	1	4.21	0.208	0.001	0.002
Red drum *vs*. Hybrid tilapia	1	1.65	0.088	0.099	0.111

*BH, Benjamini-Hochberg correction

Supplementary Table 4. (Presented in an Excel file) **ASV taxonomic classification and abundances within each sample of each fish species.** The Table includes ASVs with the abundances above 100 per ASV.

BSC, hybrid striped bass; CC, common carp; LC, European bass; MC, flathead grey mullet; RDC, red drum; TC, hybrid tilapia.
